# miR-489-3p Regulates the Oxidative Stress Response in the Liver and Gill Tissues of Hybrid Yellow Catfish (*Pelteobagrus fulvidraco*♀ × *P. vachelli*♂) Under Cu^2+^ Exposure by Targeting *Cu/Zn-SOD*

**DOI:** 10.3389/fphys.2019.00868

**Published:** 2019-07-05

**Authors:** Jun Qiang, Fanyi Tao, Wenjin Bao, Jie He, Ming Liang, Cong Liang, Haojun Zhu, Xiahong Li, Deju Chen, Pao Xu

**Affiliations:** ^1^Key Laboratory of Freshwater Fisheries and Germplasm Resources Utilization, Ministry of Agriculture, Freshwater Fisheries Research Center, Chinese Academy of Fishery Sciences, Wuxi, China; ^2^Guangdong Wulonggang Aquatic Technology Development Co., Ltd., Guangzhou, China

**Keywords:** hybrid yellow catfish (*Pelteobagrus fulvidraco*♀ × *P. vachelli*♂), Cu^2+^, miR-489-3p, Cu/Zn-SOD, antioxidant system

## Abstract

Copper/zinc superoxide dismutase (Cu/Zn-SOD) plays critical roles in protecting cells and tissues against oxidative damage. Excessive copper ions (Cu^2+^) in water can damage the cells of aquatic organisms, leading to impaired growth and development and reduced antioxidant defenses. Many regulatory factors control the response to excess Cu^2+^. Among them, microRNAs (miRNAs) are important small RNAs that regulate the expression of their target genes and participate in the oxidative stress response. In the present study, we used bioinformatics and dual luciferase reporter gene analyses to demonstrate that the miR-489-3p of hybrid yellow catfish (*Pelteobagrus fulvidraco*♀ × *P. vachelli*♂) binds to the 3′-untranslated region (UTR) of its target gene, which encodes a Cu/Zn-SOD. The regulatory relationship between this miRNA and its target gene *Cu/Zn-SOD* was analyzed using qRT-PCR and luciferase activity assays. We also investigated the effect of the loss of miR-489-3p expression on the oxidative stress response of hybrid yellow catfish exposed to Cu^2+^. The *Cu/Zn-SOD* 3′UTR region was found to be fully complementary to positions 2–9 of the 5′-end seed region of miR-489-3p. The miR-489-3p expression levels were negatively related to *Cu/Zn-SOD* expression. Silencing of miR-489-3p up-regulated *Cu/Zn-SOD* expression in the liver and gill tissues, increased activities of SOD and catalase, and reduced the malondialdehyde content. This study is the first to demonstrate that miR-489-3p targets *Cu/Zn-SOD* to mediate the oxidative response to metal stress. These findings provide a theoretical basis for further studies on the response to oxidative stress caused by metals in cultured fish, and provide an experimental basis for the management of the culture environment.

## Introduction

Copper (Cu) is a transition metal that is an essential element for growth, but it is a serious pollutant at high concentrations. Under normal circumstances, there are low Cu concentrations in surface water, but its concentrations have greatly increased in recent years because of discharges of industrial wastewater and treated urban sewage into surface water bodies. In addition, metals in solid waste such as mining waste rock and tailings can enter water bodies through leaching or weathering oxidation (Cheng et al., 2013; Alrumman et al., 2016). Copper-containing compounds are also frequently used to control fish diseases and/or as algaecides in aquaculture. Fungicides, insecticides, and fertilizers containing CuSO_4_ are widely used in agricultural production, and increase Cu pollution when surface runoff enters water bodies (Marcussen et al., 2014; Driessnack et al., 2017). In polluted aquatic ecosystems, excess copper ions (Cu^2+^) are toxic to many aquatic organisms, including algae and fish (Chen et al., 2012; Zebral et al., 2018), especially in benthic populations. Therefore, there is a greater risk of Cu poisoning in benthic species such as yellow catfish.

Reactive oxygen species (ROS) in the body regulate cell growth and cell-to-cell signaling, and also inhibit viruses and bacteria (Baldissera et al., 2018). The rates of ROS production and removal in the body are usually in a state of dynamic equilibrium (Hamanaka and Chandel, 2010). However, pathogens, xenobiotics, and/or aging can accelerate ROS formation, resulting in the accumulation of ROS to levels that cause oxidative stress via their damaging effects on biological macromolecules such as lipids, nucleic acids, and proteins. Excess ROS ultimately lead to cell damage and apoptosis (Mittal et al., 2014). Therefore, organisms have evolved a complete antioxidant defense system that maintains the balance of ROS in the body. In this system, various enzymatic and non-enzymatic antioxidants contribute to reduce the intracellular ROS concentration, thereby protecting cells and tissues from oxidative damage (Incalza et al., 2018). The Cu/zinc-superoxide dismutase (Cu/Zn-SOD) is the most widely studied type of SOD. It is mainly found in the cytoplasm of eukaryotic cells and is widely distributed among diverse organisms (Yu et al., 2017; Zeinali et al., 2018; Wang et al., 2019). In previous studies, a low concentration of Cu^2+^ was shown to increase SOD activity in the liver of *Acipenser sinensis* (Yao et al., 2010) and *Lateolabrax maculatus* (Zhu et al., 2011), whereas a high concentration of Cu^2+^ directly inhibited SOD activity. The activity of SOD in the liver of curimbata (*Prochilodus lineatus*) was shown to be stimulated by Cu^2+^ stress (dos Santos Carvalho et al., 2015). At 10% of the lethal Cu^2+^ concentration, Cu^2+^ stimulated the antioxidant responses (increased SOD activities) of common carp (*Cyprinus carpio*) and gibel carp (*Carassius auratus gibelio*) within 96 h; in rainbow trout (*Oncorhynchus mykiss*), however, increased SOD activity in the gill was found after 3 days of Cu^2+^ stress (De Boeck et al., 2004). Therefore, the SOD response to Cu^2+^ may differ among different organs and different fish species. Besides metals, other exogenous factors (such as bacterial infections and hypoxia) can stimulate SOD activity (Zhang et al., 2011; Chakravarthy et al., 2012). These findings suggested that SOD has important roles in the stress response and can be used as a marker of the antioxidant system in fish (Dogan et al., 2014).

microRNAs (miRNAs) are a class of endogenous small molecule non-coding RNAs approximately 22 nt in length that are involved in almost all cellular processes. Recent studies have shown that miR-489 expression is down-regulated in various tumor tissues, and this inhibits the expression of its target genes, such as *HER2, AKT3*, and *SPIN1*. Thus, miR-489 participates in various pathologies such as the proliferation, apoptosis, invasion, and metastasis of tumor cells (Sun et al., 2017). These processes are closely related to tumor resistance, occurrence, and development.

Yellow catfish is an important freshwater aquaculture species in China. Its production reached 417,300 tons in 2016, and grew by 20% in several consecutive years prior to 2016 (China Fishery Statistical Yearbook, 2016). Our research team began to breed hybrid yellow catfish (*Pelteobagrus fulvidraco*♀ × *P. vachelli*♂) in 2014. The progeny of the hybrids showed good growth and stress resistance, emphasizing the advantages of hybridization (Zhang et al., 2019). In our previous study, we studied the fertilization, hatching, and growth of hybrid yellow catfish (Qiang et al., 2019). We also analyzed the miRNA expression library of hybrid yellow catfish under high-fat stress. Those analyses revealed another miRNA, miR-489-3p, in the stem near the 3′ end of the miR-489 hairpin precursor. The sequence of miR-489-3p shows a high degree of homology to miR-489-3p in aca (*Anolis carolinensis*) (data from miRBase 22.0 database^1^). These miRNAs may function in the same or a similar way to their counterpart in mammals. The expression of miR-489-3p was found to be significantly increased in AT-ATX tumor-bearing mice. Exogenous addition of miR-489-3p significantly reduced the expression of its target gene MEK1 in normal cells (Kuppa et al., 2018). When endoderm-specific miR-489-3p was overexpressed in human embryonic stem cells, endoderm differentiation was accelerated and increased (Ishikawa et al., 2017).

In our previous study, we found that miR-489-3p was significantly down-regulated in the liver of hybrid yellow catfish under high-fat stress. We conducted bioinformatics analyses using TargetScan 5.2^2^ and the miRanda v3.3a toolbox^3^ with the default parameters and cut-offs (Score S ≥ 140; Energy E ≤ -7.0 Kcal/mol) (Betel et al., 2010), and screened a transcriptional library of mRNAs under high-fat stress. Those analyses indicated that a *Cu/Zn-SOD* gene [high score (142) and low energy (-20.8 Kcal/mol)] that was significantly up-regulated under high-fat stress may be a target gene of miR-489-3p. Therefore, the present study had two main aims: (1) to verify the binding site and regulatory relationship between miR-489-3p and *Cu/Zn-SOD*; and (2) to analyze the regulatory function of miR-489-3p under Cu^2+^ exposure. The results of this study shed light on the regulation of SOD under Cu^2+^ exposure, and provide important information about how fish respond to oxidative stress induced by Cu^2+^ exposure.

## Materials and Methods

### Ethical Approval

The study protocols were approved by the Freshwater Fishery Research Centre (FFRC) of the Chinese Academy of Fishery Sciences (Wuxi, China). The hybrid yellow catfish were maintained in well-aerated water and anesthetized by injection with 0.01% tricaine methanesulfonate (Sigma, St Louis, MO, United States). The tissues were sampled based on the Guide for the Care and Use of Laboratory Animals in China.

### Experimental Fish

Hybrid yellow catfish were used in these experiments. Three-year-old *P. vachelli*♀ and Pearl River wild *P. fulvidraco*♂ were used as broodstock. These fish were disease-free, strong, and had a normal body shape and color. Mixed oxytocin was used to promote maternal ovarian maturation and ovulation in the broodstock. Artificial insemination was carried out by means of wet fertilization, and the fertilized eggs were hatched by hydrostatic aeration. The incubation temperature was 28°C, the dissolved oxygen concentration in the hatching water was >6 mg⋅L^-1^, and the incubation time of the fertilized eggs was about 48 h. The newly hatched larvae were collected and placed in a breeding pond. Zooplankton were supplied as open bait for 3–5 days, and artificial powder was gradually fed for 5–10 days. After 20 days of culture, the fish were transferred to an outdoor cement pond for cultivation to the required size. The fish were fed with a normal diet (39% protein and 8% lipid) during acclimatization.

### Tissue Expression Analysis of miR-489-3p Target Gene in Normal Group and Cu^2+^-Stressed Group

The muscle, liver, kidney, spleen, blood, and gill tissues of eight healthy hybrid yellow catfish (average weight 30.3 ± 1.5 g) were selected for these analyses. A stock solution was prepared with double-distilled water and CuSO_4_.5H_2_O, an analytically pure product. This stock solution was diluted as required. In a preliminary experiment, we supplied Cu^2+^ at different concentrations (35 μg⋅L^-1^, 70 μg⋅L^-1^, 140 μg⋅L^-1^, and 280 μg⋅L^-1^) to impose Cu^2+^ stress on hybrid yellow catfish. Each treatment had 10 fish. None of the fish in the 35 μg⋅L^-1^ and 70 μg⋅L^-1^ groups died within 96 h, but the mortality rates were 10% in the 140 μg⋅L^-1^ group and 70% in the 280 μg⋅L^-1^ group. Therefore, we selected the 140 μg⋅L^-1^ Cu ^2+^ concentration to impose stress in these experiments. The Cu^2+^ concentration in water was determined by inductively coupled plasma mass spectrometry (ICP-MS). Eight hybrid yellow catfish (average weight 30.3 ± 1.5 g) were kept in 140 μg⋅L^-1^ Cu^2+^ aqueous solution (measured value, 140 ± 5 μg Cu⋅L^-1^) for 24 h, and the above tissues were selected to analyze the transcript levels of *Cu/Zn-SOD*. The fish were fed with a normal diet during the stress treatment. The water temperature was kept at 27 ± 1°C, the oxygen concentration was maintained at >6.0 mg⋅L^-1^, the ammonia, nitrate, and nitrite levels were <0.1 mg⋅L^-1^, and the pH was 7.6 ± 0.2.

### Analysis of Binding Site and Regulatory Relationship Between miR-489-3p and Its Target Gene

#### Analysis of Binding Site Between miR-489-3p and Its Target Gene

The 3′-untranslated region (UTR) of the *Cu/Zn-SOD* gene including the miR-489-3p binding target was amplified by PCR. The template was DNA extracted from the liver tissue of hybrid yellow catfish. The size of the amplified product was 409 bp. The PCR amplification product of the *Cu/Zn-SOD* 3′UTR was subcloned into the plasmid PGL3-control vector (Promega, Madison, WI, United States) by *Xba* I and *Fse* I digestion and T4 ligase, and the vector was then transformed into competent *Escherichia coli* DH5α cells. The presence and correct sequence of the gene vector was confirmed by PCR amplification and gene sequencing. The successfully identified vector was named pGL-*Cu/Zn-SOD* 3′UTR (wild-type) (wt). The six-base sequence of the miRNA-binding site was mutated to construct pGL-Cu/Zn-SOD (Mut) (mutated). To construct the pGL-*Cu/Zn-SOD* mutant, six base pairs (5′-AUGAUG-3′) in the *Cu/Zn-SOD* 3′UTR region were deleted and six new base pairs (5′-CTACTT-3′) were inserted. A scrambled miRNA mimic (5′-UUUGUACUACACAAAAGUACUG-3′) with no homology to the *Danio rerio* genome was used as the negative control (NC). To verify the binding site between the miRNA and target gene, HEK-293T cells in 96-well plates were co-transformed with pGL-*Cu/Zn-SOD* 3′UTR (wt) or pGL-Cu/Zn-SOD (Mut) and a miRNA mimic or miRNA NC using Lipofectamine 2000 transfection reagent (Invitrogen, Carlsbad, CA, United States). After 36 h, the dual-luciferase reporter gene (firefly and Renilla) was detected as described by Qiang et al. (2017b). Firefly luciferase activity was normalized against Renilla luciferase activity.

Liver tissue was dissected from healthy hybrid yellow catfish under aseptic conditions. The liver cells were isolated and purified according to the method of Chen et al. (2014), and then kept at 28°C under 5% CO_2_. When the cultured hepatocytes had grown by 80–90%, 2 μg target plasmid pEGFP-C1-3Flag-Cu/Zn-SOD-3′UTR and 50 nM or 100 nM miR-489-3p mimic or the same dose of miR-489-3p NC were co-transfected into hepatocytes using Lipofectamine 2000. The cell culture plates were observed under a fluorescence inverted microscope (Olympus X71, Olympus, Tokyo, Japan) at 48 h after transfection. Hepatocytes treated for 48 h were collected and RNA was extracted for analysis of *Cu/Zn-SOD* gene transcript levels.

#### Analysis of Regulatory Relationship Between miR-489-3p and Its Target Gene

To analyze the relationship between miR-489-3p and its target gene *in vivo*, an miR-489-3p antagomir (5′-TGTAGTATACATGCCGACGA-3′) was synthesized (RiboBio, Guangzhou, China). In total, 270 juveniles (average weight 15.5 ± 0.8 g) were distributed among nine 600-L tanks (30 fish/tank). Dry powdered miR-489-3p antagomir or the NC was dissolved in PBS (5 nmol per 50 μL PBS) (RiboBio, Guangzhou, China). The mixture was shaken thoroughly, left to stand for 5 min, and centrifuged at 5000 *g* for 15 s to prepare the working solution for injection. Using a 0.15-mL glass needle, the fish were injected via the tail vein with 30 μL miR-489-3p antagomir or the same volume of PBS or miR-489-3p NC. After injection, the needle was slowly withdrawn from the tail vein, and the injection site was gently pressed for 20 s to stop bleeding. This prevented the injected antagomir from flowing out of the injection site. Fish in the control group were injected with PBS. The livers of three fish each tank were sampled at 0, 12, 24, and 36 h after injection, immediately frozen in liquid nitrogen, and stored at -80°C until qRT-PCR analyses.

### Analysis of Oxidative Stress in Hybrid Yellow Catfish Under Cu^2+^ Exposure

In total, 360 hybrid yellow catfish juveniles (average size, 15.5 ± 0.8 g) were distributed among nine 800-L tanks (40 fish/tank). The synthesized miRNA antagomir fragment and the miRNA NC were dissolved in PBS and injected via the tail vein at a dose of 50 mg⋅kg^-1^ body weight. Fish injected with PBS served as the control. At 12 h after injection, the treated hybrid yellow catfish were exposed to 140 μg⋅L^-1^ Cu^2+^ aqueous solution for 96 h. The concentration of Cu^2+^ in the water was adjusted every 12 h, and a 1/4 volume of new water was added every day. At 0, 12, 24, 36, 60, and 108 h after injection, three fish were randomly selected from each tank, and the livers and gills were collected. The liver and gill tissues were rinsed with ice-cold saline after excision. After blotting with filter paper, they were immediately frozen in liquid nitrogen, and stored at -80°C until analyses of *Cu/Zn-SOD* gene expression and enzyme activities. The experimental management and rearing conditions were same as those described in section “Tissue Expression Analysis of miR-489-3p Target Gene in Normal Group and Cu^2+^-Stressed Group”.

### Measurement Indices

#### miR-489-3p Expression

The specific primers used to amplify miR-489-3p are shown in Table 1. Reverse transcription of miRNA cDNA was performed using the Mir-XTM miRNA First-Strand Synthesis Kit (TaKaRa, Dalian, China). For qRT-PCR amplification of miRNAs, the reaction mixture contained 10 μL 2 × SYBR Premix ExTaq^TM^ II (TaKaRa), 0.4 μL 50 × ROX reference dye, 0.4 μL upstream and downstream primers (10 μmol⋅L^-1^), 2 μL miRNA-cDNA template, and ddH_2_O to complete the volume to 20 μL. The reference gene for miRNA quantification was U6 (TaKaRa) (Qiang et al., 2017b). Three replicates were analyzed for each sample and non-template cDNA was used as a NC. The analyses were conducted using a 7900HT Fast Real-Time PCR system (Applied Biosystems, Foster City, CA, United States) with the following thermal cycling conditions: 95°C for 30 s; 95°C for 5 s, and 60°C for 31 s (40 cycles). The dissolution profile of the amplified product was analyzed at the end of each PCR cycle. After amplification, the temperature was raised from 60°C and the specificity of the amplification product was verified from the dissolution curve.

**Table 1:** Primer sequences.

miRNA or mRNA	Sequence
miR-489-3p	TGACATCATATGTACGGCTGCT
Cu/Zn-SOD	F: 5′-CACCAACGGCTGTATGAGTG-3′
	R: 5′-CCGATGATAGAGTGGGGTCC-3′
18S rRNA	F:5′-TTCCACCTCTTTCTCAACCAT-3′
	R: 5′-GGCCGTTCTTAGTTGGTGGA-3′


#### Analysis of *Cu/Zn-SOD* Gene Expression

The specific primers used to amplify the *Cu/Zn-SOD* gene are shown in Table 1. The RT reaction and qRT-PCRs of *Cu/Zn-SOD* were conducted using PrimeScript^TM^ RT Master Mix and the SYBR^®^Premix Ex Taq kit (TaKaRa) as described previously (Qiang et al., 2017b). The mRNA and *18S rRNA* primers were synthesized by Shanghai GeneCore Bio Technologies Co., Ltd., (Shanghai, China). The expression levels of miRNA and mRNA were calculated using the 2^-ΔΔCt^ method, and were analyzed with Relative Quantification Manager software.

#### Analysis of Antioxidant Enzyme Activity

The gill or liver tissue was homogenized in buffer (pH 7.4, 0.01 mol⋅L^-1^ Tris–HCl, 0.0001 mol⋅L^-1^ EDTA-Na_2_, 0.01 mol⋅L^-1^ sucrose, and 0.8% NaCl) with a homogenizer at 15,000 g. The homogenate was centrifuged at 4°C for 5 min (gill tissue: 2500 *g*; liver tissue: 3000 *g*) and the supernatant was collected.

The activities of glutathione peroxidase (GSH-Px), Cu/Zn-SOD, and catalase (CAT) were determined using kits purchased from the Nanjing Institute of Bioengineering, according to the manufacturer’s instructions. The protein content was determined by the Lowry method with bovine serum albumin as the standard (Lowry et al., 1951). The activity of Cu/Zn-SOD was determined by the xanthine oxidase method. One unit of SOD activity was defined as the amount corresponding to the inhibition rate of superoxide radicals per 50 mg tissue protein in a 1-mL reaction solution, expressed as U⋅mg^-1^ (protein) (Wang et al., 2015). The activity of CAT was determined using a colorimetric method. One unit of CAT activity was defined as the amount decomposing 1 μmol H_2_O_2_ per mg protein per second, expressed as U⋅mg^-1^ (protein). The reaction temperature in the Cu/Zn-SOD and CAT activity assays was 37°C. The malondialdehyde (MDA) content was determined by the thiobarbituric acid reaction method. The GSH-Px activity was determined as described by He et al. (2015). The non-enzymatic reduction of GSH was subtracted from total reduction of GSH to obtain GSH-Px activity. One unit of GSH-Px activity was defined as the amount of enzyme per mg protein that decreased the GSH concentration in the reaction mixture by 1 μmol⋅L^-1^.

### Statistical Analyses

The relative expression levels of gene mRNAs or miRNAs were calculated assuming that mRNA or miRNA expression was consistent with the quantitative PCR amplification efficiency of the reference gene. Based on the relative expression level in the control group at 0 h, the relative expression levels of mRNA and miRNA in each experimental group were determined by the 2^-ΔΔCT^ method. Experimental data are presented as mean ± standard deviation (SD). Statistical analyses were conducted using SPSS 21.0 statistical software. First, the data were tested for normality of distribution and homogeneity of variances, and then appropriate analyses were conducted depending on the results of the tests. Differences were considered significantly different at *P* < 0.05.

## Results

### Analysis of Potential Target Gene Expression of miR-489-3p in Different Tissues of Hybrid Yellow Catfish

**FIGURE 1:**
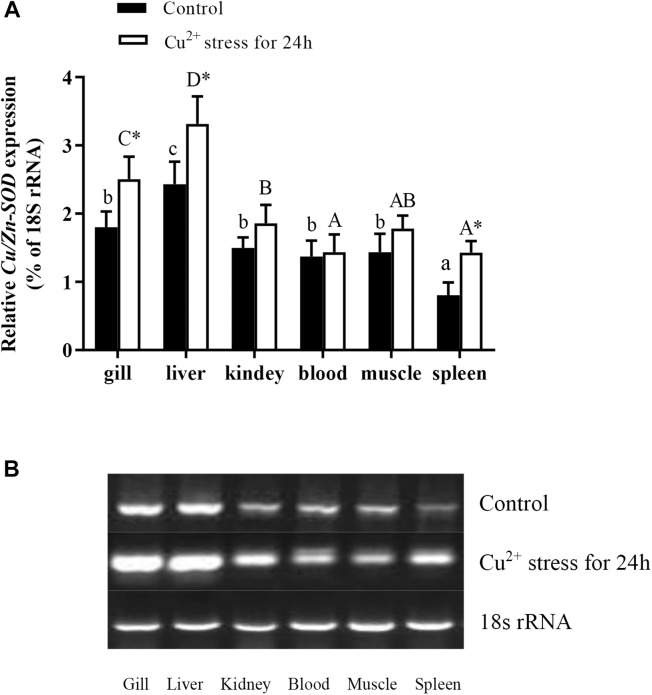
Expression analysis of *Cu/Zn-SOD* gene in different tissues of healthy hybrid yellow catfish (*Pelteobagrus fulvidraco*♀ × *P. vachelli*♂) (control) or yellow catfish subjected to 140 μg⋅L^-1^ Cu^2+^ stress for 24 h. **(A)** Expression levels of *Cu/Zn-SOD* in different tissues of control group or 140 μg⋅L^-1^ Cu^2+^-stressed group for 24 h as determined by qRT-PCR. Different lowercase letters indicate significant differences among different tissues in control group (*P* < 0.05, Duncan’s multiple comparison). Different uppercase letters indicate significant differences among different tissues in 140 μg⋅L^-1^ Cu^2+^-stressed group at 24 h (*P* < 0.05, Duncan’s multiple comparison). ^∗^ indicates significant differences between control group and 140 μg⋅L^-1^ Cu^2+^-stressed group (*P* < 0.05, independent sample *t* test). **(B)** Semi-quantitative results of *Cu/Zn-SOD* transcript levels in different tissues of control group or 140 μg⋅L^-1^ Cu^2+^-stressed group at 24 h as determined by agarose gel electrophoresis analysis.

The qRT-PCR analyses of gene expression in different tissues of healthy hybrid yellow catfish showed that *Cu/Zn-SOD* was expressed in the gill, liver, kidney, spleen, blood, and muscle, but at markedly higher levels in the liver than in other tissues (*P* < 0.05) (Figure 1). Its expression levels did not differ significantly among the gill, kidney, blood, and muscle of healthy hybrid yellow catfish. After exposure to 140 μg⋅L^-1^ Cu^2+^ for 24 h, *Cu/Zn-SOD* was expressed at higher levels in the liver and gill tissues than in other tissues, and at higher levels than before the stress treatment (*P* < 0.05). The transcript levels of *Cu/Zn-SOD* increased rapidly in the liver and gill of hybrid yellow catfish under Cu^2+^ stress. Therefore, we selected these tissues for further analyses.

### Verification of Binding Site Between miR-489-3p and Its Target Gene in Cellular Assays

**FIGURE 2:**
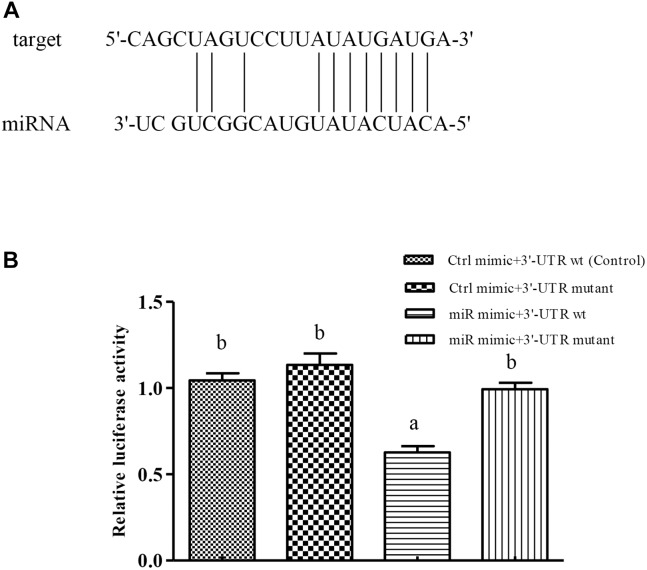
Verification of binding sites of miRNAs to potential target genes using dual luciferase reporter system. **(A)** miRNA can be paired with the 3′-UTR of potential target gene mRNA. **(B)** HEK-293T cells in 96-well plates were co-transformed with constructed pGL-Cu/Zn-SOD 3′UTR (wt) or pGL-Cu/Zn-SOD mutant (six-base mutant) and miRNA mimic or miRNA negative control (NC) (Ctrl) using Lipofectamine 2000 transfection reagent. Different lowercase letters indicate significant differences among experimental groups (*P* < 0.05, Duncan’s multiple comparison).

The *Cu/Zn-SOD* 3′UTR region (268–275 bp) was found to be fully complementary to positions 2–9 of the 5′-end seed region of miR-489-3p (Figure 2A). After validating the HEK-293T cell level, we found that the dual luciferase activity was significantly lower in the *Cu/Zn-SOD* WT + miRNA mimic group than in the other treatment groups (*P* < 0.05) (Figure 2B). These results indicated that the miRNA bound to the target gene and inhibited its translation, resulting in decreased luminescence of firefly luciferin. However, there was no significant difference in luciferase activity among miR-489-3p NC + *Cu/Zn-SOD* -3′-UTR wt (control), the miR-489-3p NC + *Cu/Zn-SOD* -3′-UTR mutant, and the miR-489-3p mimic + *Cu/Zn-SOD* -3′-UTR mutant. These results demonstrated that the miRNA was able to regulate the expression of its target gene through the binding site.

**FIGURE 3:**
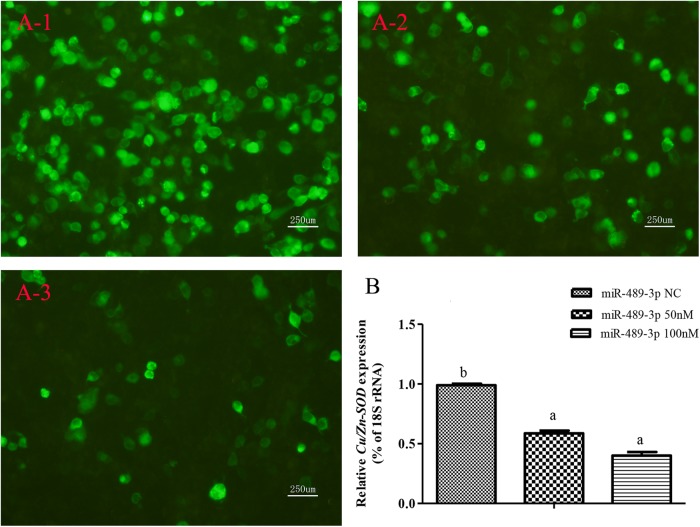
Analysis of regulatory relationships between miR-489-3p and *Cu/Zn-SOD* *in vitro*. **(A)** Luciferase activities were analyzed by co-transfecting *Cu/Zn-SOD*-3′UTR and 50 nM or 100 nM miR-489-3p mimic or same dose of miR-489-3p NC into hepatocytes. **(A-1)** pEGFP-C1-3Flag-*Cu/Zn-SOD*-3′UTR and 100 nM miR-489-3p NC were constructed and co-transfected into hepatocytes. Fluorescence was analyzed at 48 h after transfection; **(A-2)** pEGFP-C1-3Flag-*Cu/Zn-SOD*-3′UTR and 50 nM miR-489-3p mimic were constructed and co-transfected into hepatocytes. Fluorescence was analyzed at 48 h after transfection; **(A-3)** pEGFP-C1-3Flag-*Cu/Zn-SOD*-3′UTR and 100 nM miR-489-3p mimic were constructed and co-transfected into hepato cytes. Fluorescence was analyzed at 48 h after transfection; **(B)** Transcript levels of *Cu/Zn-SOD* in hepatocyte level at 48 h as determined by qRT-PCR. Different lowercase letters indicate significant differences among experimental groups (*P* < 0.05, Duncan’s multiple comparison).

When the target plasmid was transfected into hepatocytes, the luciferase activity was significantly lower in the group co-transfected with 100 nM miR-489-3p mimic than in the groups transfected with miR-489-3p NC and 50 nM miR-489-3p mimic at 48 h (*P* < 0.05) (Figure 3A). In addition, the transcript levels of *Cu/Zn-SOD* were significantly lower (*P* < 0.05) in the 50 nM and 100 nM miR-489-3p mimic groups than in the miRNA NC group, as determined by qRT-PCR (Figure 3B). These findings suggested that miR-489-3p bound to a site in the *Cu/Zn-SOD* -3′-UTR sequence.

### Analysis of Regulatory Relationship Between miR-489-3p and Its Target Gene

**FIGURE 4:**
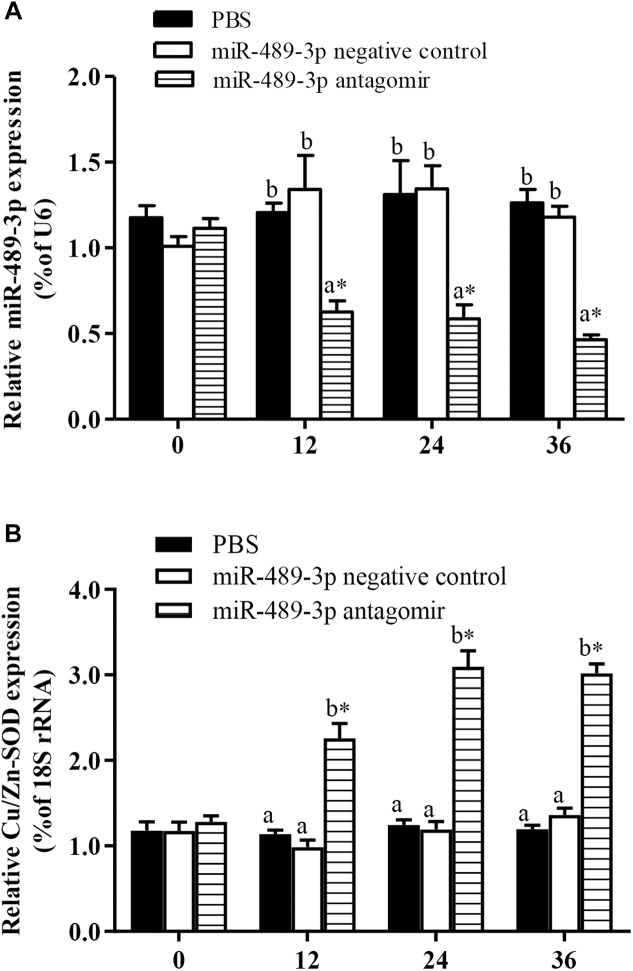
Analysis of regulatory relationships between miR-489-3p **(A)** and *Cu/Zn-SOD* **(B)** *in vivo*. Juvenile hybrid yellow catfish (*P. fulvidraco*♀ × *P. vachelli*♂) weighing about 15.5 ± 0.8 g were injected in tail vein with PBS (control), miR-489-3p (NC), miR-489-3p antagomir (dose, 50 mg⋅kg^-1^ body weight). ^∗^ indicates significant differences between values obtained before and after injection (*P* < 0.05, paired-samples *t* test). Different lowercase letters indicate significant differences among different treatments at each sampling point (*P* < 0.05, Duncan’s multiple comparison).

As shown in Figure 4, when the miR-489-3p antagomir was injected into the tail vein of hybrid yellow catfish, the expression levels of miR-489-3p in liver tissue at 12, 24, and 36 h were significantly lower than those in the control group and NC group, and the expression levels of *Cu/Zn-SOD* were significantly increased (*P* < 0.05). At each time point, the gene expression levels were not significantly different between the control group and the NC group (*P* > 0.05). These results indicated that down-regulation of miR-489-3p could significantly up-regulate the expression of *Cu/Zn-SOD* in hybrid yellow catfish liver.

### Analysis of Oxidative Stress of miR-489-3p-Mediated Target Gene in Hybrid Yellow Catfish Under Cu^2+^ Exposure

**FIGURE 5:**
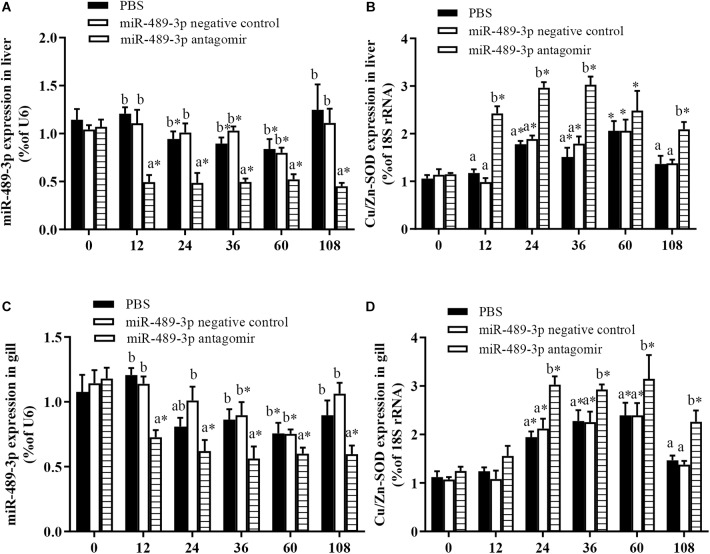
Expression levels of miR-489-3p and *Cu/Zn-SOD* gene in liver **(A,B)** and gill **(C,D)** of hybrid yellow catfish (*P. fulvidraco*♀ × *P. vachelli*♂). Juvenile yellow catfish weighing about 15.5 ± 0.8 g were injected in tail vein with PBS (control), miR-489-3p (NC), or miR-489-3p antagomir (dose, 50 mg⋅kg^-1^ body weight) and monitored for 108 h. ^∗^ indicates significant differences between values obtained before and after injection (*P* < 0.05, paired-samples *t* test). Different lowercase letters indicate significant differences among different treatments at each sampling point (*P* < 0.05, Duncan’s multiple comparison).

To further analyze the effect of miRNA-mediated *Cu/Zn-SOD* expression during the oxidative response of hybrid yellow catfish, we studied the effect of inhibition of miR-489-3p on hepatic antioxidant enzyme activity and MDA content under Cu^2+^ exposure. At 12 h, the expression levels of miR-489-3p in the livers and gills were significantly lower in the miR-489-3p antagomir treatment group than in the control group and the NC group (Figure 5A,C). The transcript level of *Cu/Zn-SOD* in the liver was significantly increased in the antagomir group, and the transcript level of *Cu/Zn-SOD* in the gill was slightly higher in the antagomir group than in the control and NC groups, but this difference was not significant (Figure 5B,D). At 12 h after injection, the treated yellow catfish were exposed to 140 μg⋅L^-1^ Cu^2+^ aqueous solution for 96 h. Within 48 h of Cu^2+^ exposure, the expression levels of miR-489-3p in the liver and gill in the control group were significantly lower than those before the stress treatment. The transcript levels of *Cu/Zn-SOD* in the liver and gill of the control group first increased and then decreased during the 96-h Cu^2+^ treatment, and were significantly higher than those before the stress treatment. The transcript levels of *Cu/Zn-SOD* in the liver and gill were significantly higher in the antagomir group than in the control group and NC group at all times during the 96-h Cu^2+^ treatment.

**FIGURE 6:**
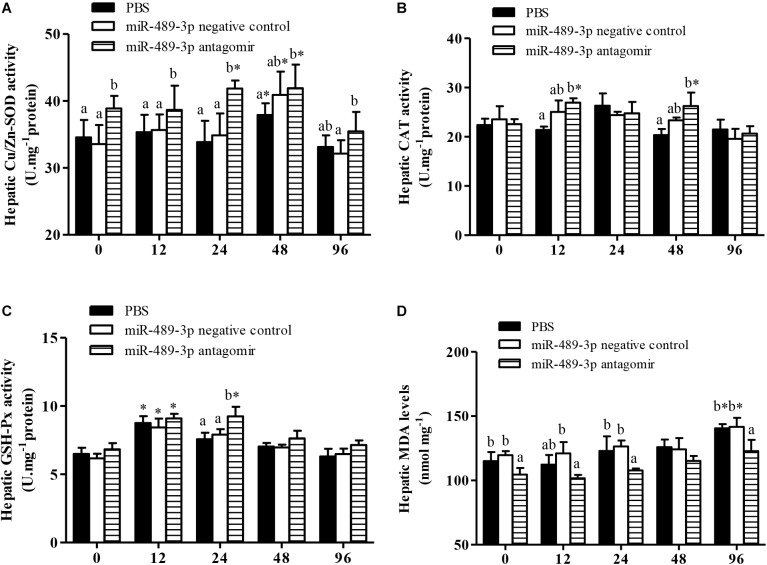
Effect of inhibition of miR-489-3p on activities/levels of hepatic Cu/Zn-SOD **(A)**, CAT **(B)**, GSH-Px **(C)**, and MDA **(D)** in hybrid yellow catfish (*P. fulvidraco*♀ × *P. vachelli*♂) exposed to Cu^2+^. At 12 h after injection with PBS (control) or miR-489-3p antagomir, yellow catfish were subjected to acute Cu^2+^ exposure for 96 h. ^∗^ indicates significant differences between values obtained before and after injection (*P* < 0.05, paired-samples *t* test). Different lowercase letters indicate significant differences among different treatments at each sampling point (*P* < 0.05, Duncan’s multiple comparison).

**FIGURE 7:**
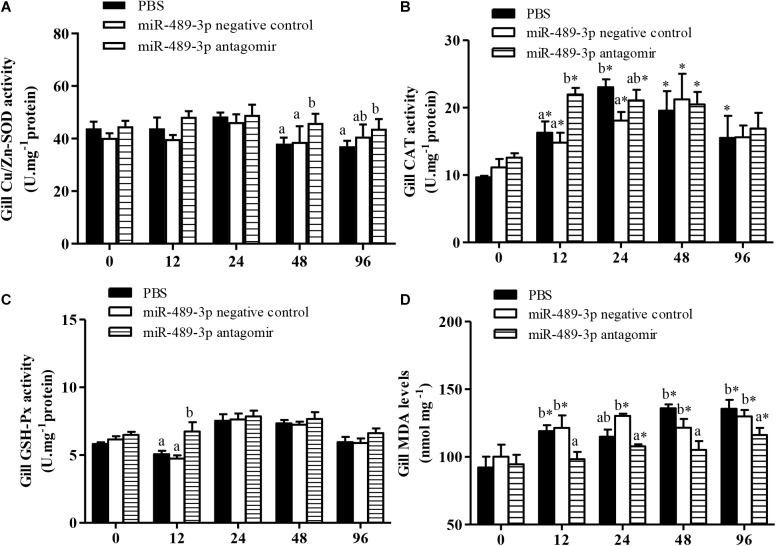
Effect of inhibition miR-489-3p on activities/levels of gill Cu/Zn-SOD **(A)**, CAT **(B)**, GSH-Px **(C)**, and MDA **(D)** in hybrid yellow catfish (*P. fulvidraco*♀ × *P. vachelli*♂) exposed to Cu^2+^. At 12 h after injection of PBS (control) or miR-489-3p antagomir, yellow catfish were subjected to acute Cu^2+^ exposure for 96 h. ^∗^ indicates significant differences between values obtained before and after injection (*P* < 0.05, paired-samples *t* test). Different lowercase letters indicate significant differences among different treatments at each sampling point (*P* < 0.05, Duncan’s multiple comparison).

In all treatment groups, the Cu/Zn-SOD activity in the liver was significantly higher at 48 h of Cu^2+^ exposure than before the stress treatment (Figure 6), but had significantly decreased by 96 h of Cu^2+^ exposure. The activities of Cu/Zn-SOD and CAT in the gill first increased and then decreased in all experimental groups (Figure 7). At 48 h of Cu^2+^ exposure, the gill SOD and hepatic CAT activities were significantly higher in the antagomir group than in the other treatment groups. The MDA contents in the liver and gill gradually increased under Cu^2+^ exposure in all treatment groups. However, the MDA contents in the liver and gill were significantly lower in the antagomir group than in the other treatment groups at 12, 24, and 96 h of Cu^2+^ exposure. The activities of GSH-Px in the gill were significantly lower in both the control group and the miRNA NC group than in the antagomir group at 12 h of Cu^2+^ exposure, but did not differ significantly among the experimental groups from 24 h to 96 h of Cu^2+^ exposure. In all experimental groups, the activity of GSH-Px in the liver first increased and then decreased during Cu^2+^ exposure, but only differed among groups at 24 h of Cu^2+^ exposure.

## Discussion

Superoxide dismutases are metalloenzymes that are widely distributed in cells of microorganisms, animals, and plants. According to the metal ions in the enzyme, SODs can be divided into three classes: Cu/Zn-SOD, Mn-SOD, and Fe-SOD (Fridovich, 1995). Different types of SODs are expressed differently in different tissues or at different stages in the same tissue. Among them, Cu/Zn-SOD was the first to be discovered, and is the most widely distributed. It has important physiological functions and medicinal prospects (Zhang et al., 2011). The capacity for Cu/Zn-SOD to scavenge O_2_^-^ is related to its gene expression level and enzyme activity. The activity of Cu/Zn-SOD in an organism tends to increase under mild stress, but decrease under severe stress. A decrease in SOD activity under severe stress can lead to excess ROS accumulation in the organism, resulting in cellular damage (Liu et al., 2015; Phull et al., 2018).

miRNAs are important small RNAs that regulate gene expression in animals and plants. They are involved in the physiological and metabolic processes during growth and development, and in stress responses, via their regulation of target gene expression. They also play important roles in the responses of plants and animals to metal stress (Wang et al., 2013; Jin et al., 2015; Noman and Aqeel, 2017). In previous studies, five miRNAs were up-regulated in the gill Chinese surf clam (*Mactra chinensis*) under cadmium (Cd^2+^) stress, and their potential target genes were involved in the regulation of ubiquitin ligase E3, the Wnt signaling pathway, and G protein signaling (Zhang J.J. et al., 2016). In genetically improved farmed tilapia (GIFT, *Oreochromis niloticus*) exposed to Cd^2+^, down-regulation of miR-122 in the liver up-regulated the expression of its target gene encoding metallothionein, which affected blood parameters, serum glucose levels, and the activities of alanine aminotransferase and aspartate aminotransferase, thus relieving stress damage in the liver (Qiang et al., 2017b).

miR-489 is located on chromosome 7 (chr7:93483936–93484019) in humans, and is highly conserved among vertebrates such as humans, macaques, mice, dogs, and elephants (Sun et al., 2017). miR-489 plays an important role in embryo development and tumor formation in invertebrates. The role of miR-489 in tumor cells has become a research hotspot in recent years. miR-489 mainly functions to suppress tumors, similar to tumor suppressor genes. It weakens the activity of its target gene, whose role is to promote tumor cell growth and proliferation. miR-489 is down-regulated in some types of tumor tissues or cells, while overexpression of miR-489 has been shown to inhibit tumor cell proliferation, invasion, and transfer (Jiang et al., 2014; Wu et al., 2014; Patel et al., 2016).

In this study, miR-489-3p in hybrid yellow catfish mediated its target gene *Cu/Zn-SOD* in the liver and gill tissues during the stress response to Cu^2+^. We used bioinformatics and dual luciferase reporter gene detection systems to verify the binding site of this miRNA to its target, *Cu/Zn-SOD*, at the cellular level. Down-regulation of miR-489-3p significantly increased the transcript levels of *Cu/Zn-SOD*. Only SODs use O_2_^-^⋅as their substrate, and they are the first antioxidant enzyme in the ROS scavenging system. Thus, they are core biological antioxidant enzymes (dos Santos Carvalho et al., 2015). In healthy hybrid yellow catfish, the transcript levels of *Cu/Zn-SOD* were higher in the liver and gill tissues than in other tissues. Fish gills are important for respiration and ionoregulation, and are the main surface in contact with the environment. The oxidative phosphorylation process of aerobic respiration produces excessive O_2_^-^⋅, which induces Cu/Zn-SOD under stress. In this study, the transcript level of *Cu/Zn-SOD* in the gill tissue of hybrid yellow catfish was significantly increased by 48 h of exposure to 140 μg⋅L^-1^ Cu^2+^. The elevated *Cu/Zn-SOD* transcript level may have led to increased levels of Cu/Zn-SOD protein. Fish can show a typical “poisonous excitatory effect” in the early stages of contaminant exposure (Stebbing, 1982). However, this effect can disappear with prolonged exposure time because of the continuous production of ROS in response to the pollutant. This can lead to damaged physiological function, as observed in yellow catfish. The Cu/Zn-SOD activity in the gills of the control group was significantly inhibited after 96 h of Cu^2+^ exposure. The up-regulated *Cu/Zn-SOD* transcript level in the antagomir group may have helped to maintain Cu/Zn-SOD activity at a higher level. With exposure to Cu^2+^ for 96 h, the gene expression levels and enzyme activity of Cu/Zn-SOD in the gills were significantly higher in the antagomir group than in the control group. This increased the decomposition of ROS and reduced stress damage in the antagomir group.

The liver of aquatic animals is an important site not only for the metabolism and storage of various biological macromolecules, but also for detoxification. The liver is the main site of metal accumulation in aquatic animals and one of the most strongly affected organs by metal toxicity (Zhang Q.F. et al., 2016). The transcript level of *Cu/Zn-SOD* and Cu/Zn-SOD enzyme activity in the liver tissues were significantly higher in the miR-489-3p antagomir group than in the control group after Cu^2+^ exposure. After the miRNA antagomir was injected into the tail vein of yellow catfish, the antagomir was able to enter the liver tissue via blood flow, so it could directly regulate its complementary miRNA, and consequently, its target gene, in the liver. In our study, the regulatory effect of the miRNA antagomir was stronger in liver tissue than in gill tissue. The higher *Cu/Zn-SOD* transcript level and Cu/Zn-SOD enzyme activity in the antagomir group may have contributed to relieving oxidative stress in the liver. Hansen et al. (2006) found that *SOD* expression in brown trout (*Salmo trutta*) was enhanced during chronic exposure to various metals, and the sensitivity to oxidative stress, especially in the liver, was greater under Cu^2+^ exposure. The adaptability of brown trout to metal stress was proposed to result from domestication, rather than inheritance. Qiang et al. (2017b) reported similar trends in hepatic SOD activity in GIFT in response to Cd^2+^ exposure. In future studies, we intend to study the regulation network and mechanism in fish exposed to metals by analyzing *SOD*-overexpression and knock-out lines.

Under normal circumstances, the production and elimination of ROS in organisms are in a state of dynamic equilibrium. To maintain ROS homeostasis, organisms have evolved an effective antioxidant system. In addition to SOD, GSH-Px and CAT remove excess ROS from the body, and play an important role in reducing the oxidative damage caused by external stress (Qiang et al., 2017a). In this experiment, CAT activities in the liver and gill tissues of hybrid yellow catfish significantly increased during the early stage of Cu^2+^ exposure, but began to decrease with prolonged exposure time, similar to the results reported by Qiang et al. (2017b). In other studies, CAT activity increased in the liver of zebrafish (*D. rerio*) under acute Cd^2+^ exposure, and changes in CAT activity differed significantly between male and female fish (Xie et al., 2018). The activity of CAT in the liver of *Sparus aurata* and *C. carpio* increased in response to low doses of Cd^2+^, but decreased in response to high Cd^2+^ doses (Jia et al., 2011; Souid et al., 2013). In *Perna canaliculus*, CAT activities in the gills, digestive gland, and blood lymphocytes were also elevated under acute or sub-chronic Cd^2+^ exposure (Chandurvelan et al., 2013). In the present study on hybrid yellow catfish, the response of CAT activity in the gill was stronger than that in the liver. The greater response of CAT activity in the gill than in the liver could be because of differences in the extent of Cu^2+^ exposure between the tissues, and/or the presence of different isoforms with different metal sensitivities in the two tissues. The temporal dynamics of the responses of single antioxidant enzymes to chemical pollutants is a complex phenomenon, with high species-, tissue-, and exposure-specificity. We speculated that when a small amount of Cu^2+^ entered the fish body, ROS were produced. The increase in hepatic CAT activity contributed to the decomposition of H_2_O_2_. However, as Cu^2+^ accumulated in the body, excessive ROS blocked the synthesis of CAT in the liver and gill tissues, resulting in decreased enzyme activity.

Another enzyme that decomposes H_2_O_2_ is GSH-Px, which is an important component of the antioxidant system. This enzyme helps to protect the integrity of cell membranes and related functions in aquatic organisms (Lin et al., 2018). There were differences in GSH-Px activity between the liver and the gill of the wild-type yellow catfish exposed to Cu^2+^ for 96 h. The GSH-Px activity in the gills was significantly inhibited at 12 h of Cu^2+^ exposure, indicating that the gill cells may have been damaged to some extent. With prolonged Cu^2+^ exposure, large amounts of ROS were generated, the body’s stress system and antioxidant defense system were fully activated, and the activity of GSH-Px gradually increased to reduce damage caused by ROS. The GSH-Px in the liver responded to Cu^2+^ stress faster than did the GSH-Px in the gill. At 12 h of Cu^2+^ exposure, higher GSH-Px activity helped to reduce ROS and alleviate cellular oxidative stress. Compared with the wild-type group, the antagomir group showed higher *Cu/Zn-SOD* transcript levels in the liver and gill tissues. This increase, as well as the higher CAT and GSH-Px activities in the antagomir group, may have increased their antioxidant capacity.

When the fish body is exposed to a contaminated environment for a long time, a large amount of accumulated ROS will induce lipid peroxidation in the cell membranes and produce MDA, one of the main markers of cellular oxidative damage. During the 96-h exposure to Cu^2+^, the MDA content in the liver and gill tissues of the wild-type group tended to increase, indicating that Cu^2+^ induced lipid oxidation in both hepatocytes and gill cells. Similar results have reported in studies on other fish such as *Channa punctata*, *Paralichthys olivaceus*, and *Gobiocypris rarus* (Pandey et al., 2008; Cao et al., 2010; Wu et al., 2019). The enhanced antioxidant enzyme activities in the liver and gill tissues of the wild-type group at 48 h of exposure to Cu^2+^ alleviated oxidative damage; however, damage to the liver and gill tissues had increased by 96 h of Cu^2+^ exposure. Excess MDA can cross-link with proteins, and this destroys the structure and function of enzymes, including antioxidant enzymes. Damaged antioxidant enzymes cannot remove excess ROS, leading to a sharp rise in MDA content, and ultimately to fish death. Compared with the wild-type group, the miR-489-3p antagomir group showed higher SOD and CAT activities, and this may have resulted in greater elimination of excess ROS and alleviation of oxidative damage in cells.

## Conclusion

This is the first report of the function of miR-489-3p in hybrid yellow catfish. Our results showed that this miRNA binds to the 3′-UTR end of its target gene, *Cu/Zn-SOD*, to negatively regulate its expression. Silencing of miR-489-3p significantly enhanced *Cu/Zn-SOD* expression in the liver and gill tissues of fish under Cu^2+^ exposure, and improved the antioxidant capacity, thus alleviating cellular oxidative damage. The results of this study provide new perspectives on molecular regulation in fish exposed to metal pollution. In further research, we intend to use I-cell stage fertilized eggs or hepatocytes of yellow catfish for experiments on this miRNA by overexpression or knockout of its target gene. The aim of such studies is to reveal the regulation and transmission networks of the miRNA-mediated response to metal stress in fish via alterations in gene transcript and protein levels *in vivo*.

## Author Contributions

PX and JQ conceived and designed the experiments. ML and CL cultured hybrid yellow catfish. FT and WB carried out the experiments. JH and FT collected samples, extracted RNA, and conducted qRT-PCR experiments. DC and XL measured biochemical parameters. HZ analyzed the data. JQ wrote the manuscript with contributions from all other authors. All authors read and approved the final version of the manuscript.

## Conflict of Interest Statement

ML and CL are employed by Guangdong Wulonggang Aquatic Technology Development Co., Ltd. The remaining authors declare that the research was conducted in the absence of any commercial or financial relationships that could be construed as a potential conflict of interest.

## Footnotes

1 http://www.mirbase.org/

2 http://www.targetscan.org/vert_50/

3 http://www.microrna.org/microrna/home.do
